# Clinical Significance of CD90(+) Circulating Tumor Cells as Dynamic Biomarkers in Unresectable Hepatocellular Carcinoma Treated with Atezolizumab/Bevacizumab and Lenvatinib

**DOI:** 10.3390/cancers17172829

**Published:** 2025-08-29

**Authors:** Takuto Nosaka, Yosuke Murata, Yu Akazawa, Tomoko Tanaka, Kazuto Takahashi, Tatsushi Naito, Masahiro Ohtani, Yasunari Nakamoto

**Affiliations:** Second Department of Internal Medicine, Faculty of Medical Sciences, University of Fukui, Fukui 910-1193, Japan; yosukem@u-fukui.ac.jp (Y.M.); aka0124@u-fukui.ac.jp (Y.A.); kawakami@u-fukui.ac.jp (T.T.); tkazuto@u-fukui.ac.jp (K.T.); naitot@u-fukui.ac.jp (T.N.); mohtani@u-fukui.ac.jp (M.O.)

**Keywords:** hepatocellular carcinoma, circulating tumor cell, CD90, tumor markers, atezolizumab plus bevacizumab

## Abstract

*Background:* Atezolizumab plus bevacizumab and lenvatinib are standard treatments for advanced hepatocellular carcinoma, but conventional tumor markers such as alpha-fetoprotein and des-gamma-carboxy prothrombin have a limited ability to reflect treatment responses. Circulating tumor cells with cancer stem cell characteristics have emerged as promising biomarkers. We examined the dynamics of cancer stem cell-related circulating tumor cell subsets and tumor markers at early and maximal response phases in patients with unresectable hepatocellular carcinoma undergoing systemic therapy. *Methods:* Sixty-two patients treated with either atezolizumab plus bevacizumab or lenvatinib were retrospectively analyzed. Peripheral blood was collected at baseline, during the early phase (during one to three months), and at maximal response. Circulating tumor cell subsets expressing cancer stem cell markers (CD90, epithelial cell adhesion molecule; CD133, vimentin) were assessed using multiparametric flow cytometry and compared with alpha-fetoprotein and des-gamma-carboxy prothrombin. *Results:* Early decreases in CD90-positive circulating tumor cells after therapy were associated with tumor shrinkage, longer periods of progression-free survival in both groups, and prolonged overall survival in the atezolizumab plus bevacizumab group. By contrast, early changes in alpha-fetoprotein and des-gamma-carboxy prothrombin were not consistently related to tumor size, progression-free survival, or overall survival. At maximal response, changes in CD90-positive circulating tumor cells reflected tumor burden more accurately than alpha-fetoprotein or des-gamma-carboxy prothrombin. *Conclusions:* These findings indicate that cancer stem cell-related circulating tumor cell subsets, particularly CD90-positive cells, may serve as valuable biomarkers for monitoring treatment response and predicting prognosis in unresectable hepatocellular carcinoma. CD90-positive circulating tumor cells perform dynamic monitoring superior to conventional markers such as alpha-fetoprotein and des-gamma-carboxy prothrombin.

## Simple Summary

Atezolizumab plus bevacizumab and lenvatinib are standard treatments for unresectable hepatocellular carcinoma; however, tumor markers such as alpha-fetoprotein and des-gamma-carboxy prothrombin have a limited ability to reflect treatment responses. Circulating tumor cells are non-invasive biomarkers associated with cancer stemness and treatment resistance. We assessed circulating tumor cell subsets expressing cancer stem cell markers (CD90, epithelial cell adhesion molecule, CD133, vimentin) using multiparametric flow cytometry at early and maximal response phases in patients receiving atezolizumab plus bevacizumab or lenvatinib. Early decreases in CD90-positive circulating tumor cells after therapy initiation were associated with tumor shrinkage and longer progression-free survival in both groups, as well as prolonged overall survival in the atezolizumab plus bevacizumab group. At maximal response, changes in CD90-positive circulating tumor cells reflected tumor burden more accurately than alpha-fetoprotein or des-gamma-carboxy prothrombin. These findings highlight the potential of CD90-positive circulating tumor cells to become dynamic biomarkers in systemic therapy for unresectable hepatocellular carcinoma.

## 1. Introduction

Hepatocellular carcinoma (HCC) is the third-most common cause of cancer-related deaths worldwide and is the leading cause of mortality among patients with liver cirrhosis [1,2]. Advancements in our understanding of tumor biology have substantially transformed systemic therapy for unresectable hepatocellular carcinoma in cases where surgical resection or local therapy is not feasible. Molecular targeted agents and immune checkpoint inhibitors (ICIs) have become the cornerstone of treatment, leading to remarkable improvements in overall survival [3].

The combination therapy of atezolizumab, an immune checkpoint inhibitor, and bevacizumab, an anti-vascular endothelial growth factor antibody (Atezo + Bev), demonstrated efficacy in the IMbrave150 trial and has been established as the standard first-line treatment for unresectable HCC [4]. In addition, lenvatinib, a multikinase inhibitor, has also become widely utilized as a first-line therapy for unresectable HCC [5]. However, despite the expansion of these therapeutic options, a reliable biomarker capable of assessing treatment response in real time has yet to be established. The conventional tumor markers alpha-fetoprotein (AFP) and des-gamma-carboxy prothrombin (DCP) have limitations in reflecting therapeutic responses and clinical status efficiently due to their non-specificity [6,7]. They are also ineffective in the presence of tumors that do not produce these marker proteins.

Circulating tumor cells (CTCs), which are tumor cells shed from primary tumors into the bloodstream, are closely associated with tumor invasiveness, metastatic potential, stemness, and treatment resistance [8]. As a non-invasive liquid biomarker, CTCs are expected to be useful for monitoring treatment efficacy and predicting prognosis [9,10]. Subsets of CTCs expressing cell surface markers such as CD90 [11,12], EpCAM [13], CD133 [12], and vimentin [12] have been associated with cancer stemness and epithelial–mesenchymal transition (EMT), and reports on these subsets are increasing. We previously reported that changes in the number and protein expression levels of CTCs expressing CD90 and EpCAM correlated with treatment efficacy in patients with HCC undergoing Atezo + Bev treatment [14]. However, it remains unclear whether CTCs offer superior assessment of disease status compared to AFP and DCP, which are tumor markers commonly monitored in clinical practice.

In this study, we analyzed the dynamics of CTC subsets expressing CSC markers at both early treatment stages and at the time of maximal therapeutic response in patients with unresectable HCC treated with Atezo + Bev or lenvatinib. Furthermore, we compared these findings with the tumor markers AFP and DCP. Among the CTC subsets, changes in CD90(+) CTC counts were significantly associated with alterations in tumor size, progression-free survival (PFS), and overall survival (OS). In contrast, early changes in AFP and DCP levels showed no association with changes in tumor size. In the treatment of unresectable HCC with Atezo + Bev or lenvatinib, the dynamics of peripheral blood CD90(+) CTCs were shown to be strongly associated with tumor responsiveness and prognosis, suggesting their potential as valuable biomarkers.

## 2. Materials and Methods

### 2.1. Study Protocol and Patients

**Figure 1:**
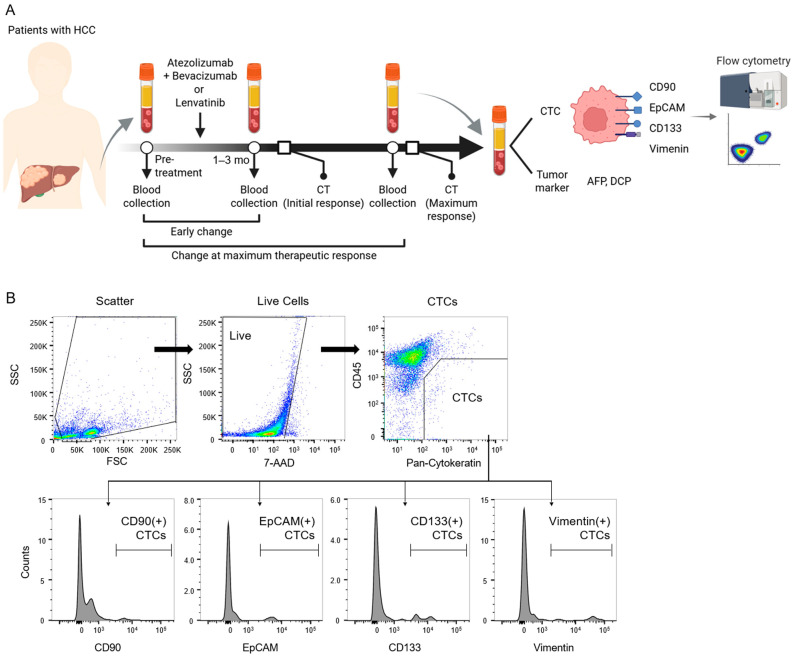
Study design and comparative workflow of CTCs and tumor markers. (**A**) Study design schematics. Peripheral blood was collected from patients with unresectable hepatocellular carcinoma before treatment, at 1–3 months after starting Atezo + Bev or lenvatinib therapy (early change), and at the maximal tumor response stage (maximal response). CTCs were isolated and analyzed via flow cytometry for cancer stem cell markers (CD90, EpCAM, CD133, and vimentin) and compared with levels of AFP and DCP. (**B**) Schematic workflow of multiparametric flow cytometry for CTC isolation and molecular profiling: (I) dead cell exclusion with Fixable Viability Stain; (II) selection of CD45(−)/PanCK(+) cells as CTCs; and (III) assessment of CD90, CD133, EpCAM, or vimentin expression.

This retrospective study reviewed 121 patients who received either Atezo + Bev (*n* = 51) or lenvatinib (*n* = 72) at the University of Fukui Hospital between September 2020 and January 2025. The exclusion criteria were as follows: (1) incomplete clinical data or evaluation of treatment efficacy (*n* = 2), and (2) incomplete collection of CTCs during the treatment course (*n* = 67). As a result, 62 patients (Atezo + Bev, *n* = 37; lenvatinib, *n* = 25) were included in the analysis (Supplementary Figure S1). Peripheral blood samples were collected at baseline, during the early treatment phase (defined as one to three months after the initiation of therapy), and at maximal therapeutic response (defined as the time of maximal change in tumor size) (Figure 1A). The clinical characteristics of the patients are summarized in Table 1. The diagnosis of hepatocellular carcinoma was based on imaging findings according to the practice guidelines of the American Association for the Study of Liver Diseases or a histological examination via liver tumor biopsy. This study was conducted in accordance with the Declaration of Helsinki and was approved by the Research Ethics Committee of the Faculty of Medicine, University of Fukui (Approval No.: 20200086; Approval Date: 20 August 2020). Written informed consent was obtained from all participants.

### 2.2. Etiological Background of Liver Disease

The etiology was classified as HBV positive for patients with detectable hepatitis B surface antigen (HBsAg), and as HCV positive for those with positive hepatitis C virus antibody (HCV Ab).

### 2.3. Treatment Regimens of Atezo + Bev and Lenvatinib

Atezolizumab (1200 mg) and bevacizumab (15 mg/kg) were administered every three weeks according to the IMbrave150 protocol [4]. Atezolizumab and bevacizumab were discontinued or dose-reduced in response to adverse events (AEs). Patients received oral lenvatinib at a dose of 8 mg daily if they weighed under 60 kg, and 12 mg daily if their weight was 60 kg or above. Lenvatinib dose reduction or temporary interruption was performed if grade 2 AEs were deemed unacceptable or if grade 3 AEs occurred. Treatment selection was determined based on clinical practice guidelines and the collective expertise of multidisciplinary teams at each hospital, with the final decision made through after discussions between each clinician and the patient. Treatment was discontinued if unacceptable adverse events or disease progression (PD) were observed. Adverse events were evaluated and graded according to the National Cancer Institute Common Terminology Criteria for Adverse Events (CTCAE) version 5.0 (https://ctep.cancer.gov/protocoldevelopment/electronic_applications/ctc.htm, accessed on 23 July 2025).

### 2.4. Evaluation of Treatment Efficacy

The initial treatment response was evaluated 8 to 12 weeks after the first administration using dynamic CT or gadolinium-ethoxybenzyl-enhanced MRI (Gd-EOB-MRI). Subsequent evaluations were performed every 6 to 12 weeks. Radiological treatment response was evaluated according to the Response Evaluation Criteria in Solid Tumors (RECIST) version 1.1. Progression-free survival (PFS) was defined as the period from treatment initiation to the date of radiologically confirmed tumor progression or death. Overall survival (OS) was defined as the period from the date of treatment initiation to the date of death.

### 2.5. Enrichment of CTCs

CTCs were enriched using the methods described in our previous studies [14,15]. Peripheral venous blood samples (10 mL each) were collected into tubes containing EDTA-2Na. To avoid contamination with epithelial cells, the first 5 mL of blood was discarded. CTCs were enriched using the RosetteSep Human CD45 Depletion Cocktail (StemCell Technologies, Vancouver, NA, Canada). Samples were processed within 4 h of collection, incubated with 50 µL of Depletion Cocktail per 1 mL of blood for 20 min, and diluted with PBS (Gibco™, Waltham, MA, USA) containing 2% FBS. Centrifugation was performed at 1200× *g* for 15 min at room temperature using SepMate 50 mL tubes containing Lymphoprep™. The supernatant was transferred to a new tube, washed with PBS containing 2% FBS, and centrifuged at 300× *g* for 10 min before the cells were resuspended and collected in PBS.

### 2.6. Flow Cytometry Analysis

Enriched cells using RosetteSep were analyzed via flow cytometry, as described in our previous study [14]. Enriched cells were incubated with APC/Cyanine7 mouse anti-human CD45 (BioLegend, San Diego, CA, USA), PE mouse anti-human pan-Cytokeratin (Cayman Chemical, Ann Arbor, MI, USA), PE-Cy7 mouse anti-human CD90 (BD Biosciences, Franklin Lakes, NJ, USA), BV510 mouse anti-human CD133 (BD Biosciences), APC mouse anti-human EpCAM (BD Biosciences), Alexa Fluor 488 mouse anti-human vimentin (BD Biosciences), or isotype control mouse IgG (BD Biosciences). The antibodies used are listed in Table 2. Dead cells were identified using 7-AAD (BD Biosciences). In this study, the population characterized as 7-AAD(−)/CD45(−)/PanCK(+) was defined as CTCs. Subsets expressing CD90, an epithelial cell adhesion molecule, CD133, and vimentin were specifically evaluated (Figure 1B). These findings were compared with serum levels of AFP and DCP to assess the relative utility of CTC dynamics and conventional tumor markers. Flow cytometry was performed using BD FACS Aria II (BD Biosciences), and analyses were conducted with FlowJo version 10.10. For compensation, isotype control IgGs (BD Biosciences) and CompBeads (BD Biosciences) were used.

### 2.7. Statistical Analysis

Statistical analyses were performed using the Wilcoxon signed-rank test, the Mann–Whitney U test, or the Tukey–Kramer multiple comparison test. Cumulative survival was estimated using the Kaplan–Meier method and compared using the log-rank test. Statistical analyses were performed using GraphPad Prism version 10.5.0 (GraphPad Software Inc., San Diego, CA, USA) and EZR software (version 1.61; Saitama Medical Center, Jichi Medical University, Saitama, Japan), which is a graphical user interface for R, with *p* < 0.05 considered statistically significant. This study is an exploratory retrospective analysis, and therefore, a priori calculation of the required sample size was not performed. Instead, we estimated the detectable effect size in each treatment group using the number of observed events and Schoenfeld’s approximation, with a two-sided α of 0.05 and 80% power. Receiver-operating characteristic (ROC) curve analysis was used to assess the predictive performance of biomarkers. The optimal cutoff value was determined based on Youden’s index (sensitivity + specificity − 1).

## 3. Results

### 3.1. Early Changes in CTC Subsets and Tumor Markers as a Predictive Factor for Treatment Outcomes in Atezo + Bev Therapy

**Figure 2:**
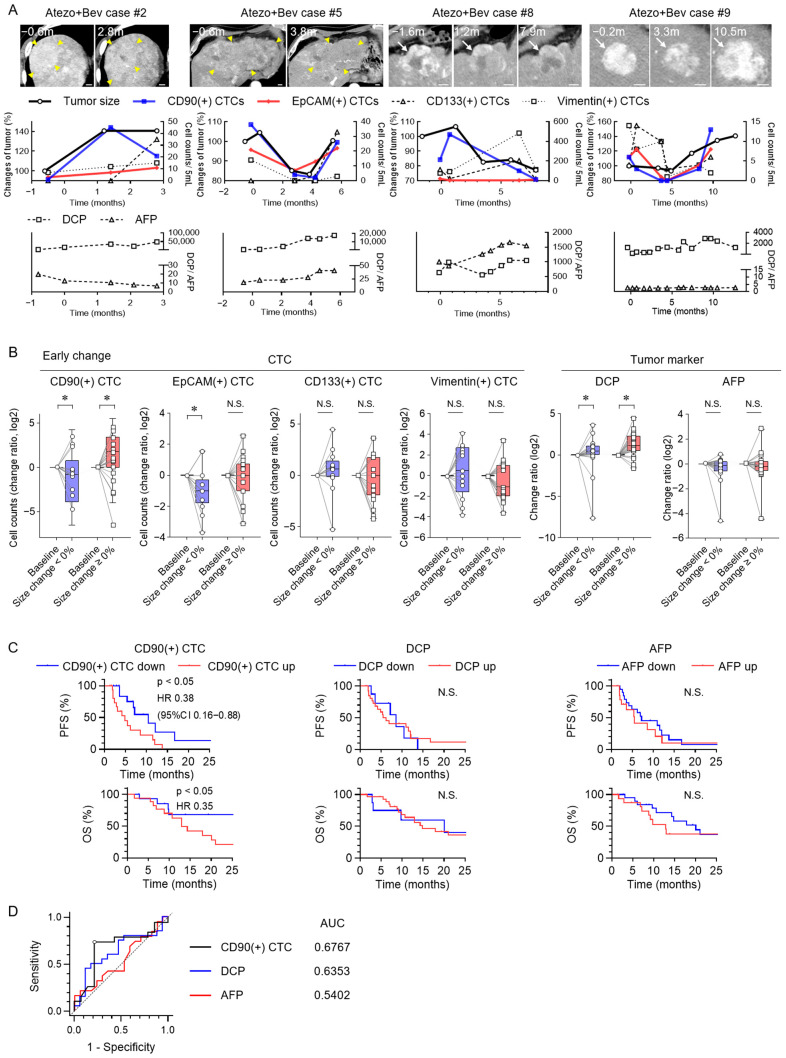
Early changes in CTC subsets and tumor markers predicting outcomes for Atezo + Bev therapy. (**A**) Representative clinical courses of four HCC patients undergoing Atezo + Bev therapy. Arterial-phase dynamic CT images indicate tumor regions (yellow arrowheads or white arrows), along with changes in CTC subsets (CD90, EpCAM, CD133, and vimentin) and tumor markers (AFP and DCP). Scale bar: 10 mm (white bar). (**B**) Early changes in CTC subsets and tumor markers in patients with HCC receiving Atezo + Bev, comparing groups that experienced tumor shrinkage (<0%) to those that experienced tumor enlargement (≥0%) from baseline. (**C**) Kaplan–Meier analysis of PFS and OS according to decreases or increases in CD90(+) CTC counts, DCP, and AFP levels during early response evaluation. (**D**) Receiver-operating characteristic (ROC) curves of ΔCD90(+) CTCs, ΔDCP, and ΔAFP (log2 ratio) for predicting early changes in tumor size. (**B**) Box plots show medians (lines), interquartile ranges (boxes), and ranges (whiskers). Wilcoxon matched-pairs test. (**C**) Log-rank test. * *p* < 0.05, AUC, area under the curve; CI, confidence interval; HR, hazard ratio; N.S., not significant.

Time-course changes in tumor size, CTC counts, and tumor markers were analyzed in four representative HCC cases treated with Atezo + Bev (Figure 2A). Early tumor shrinkage was accompanied by changes in CD90(+) and EpCAM(+) CTC counts, whereas AFP and DCP remained largely unchanged. Among the 32 cases assessed, significant variations were detected in CD90(+) CTCs, corresponding to early changes in tumor size (Figure 2B). EpCAM(+) CTCs decreased in tumor responders, and DCP increased in progressive cases. Patients with early decreases in CD90(+) CTCs had significantly longer PFS and OS than those with increased counts (Figure 2C); however, changes in AFP and DCP did not produce this association. ROC analysis of early tumor size changes showed that the AUC of CD90(+) CTCs was 0.6767 (Figure 2D). The optimal cutoff value for ΔCD90(+) CTCs was 0.0 (log2 ratio), with a sensitivity of 78.6% and a specificity of 73.7% at this threshold. The corresponding positive likelihood ratio was 2.99, and the negative likelihood ratio was 0.29. These findings indicate that early CD90(+) CTC dynamics reflect tumor size changes and are predictive of survival in HCC patients receiving treatment with Atezo + Bev.

### 3.2. Correlation of CTC Subset and Tumor Marker Dynamics with Tumor Size at Maximal Response for Atezo + Bev Treatment

**Figure 3:**
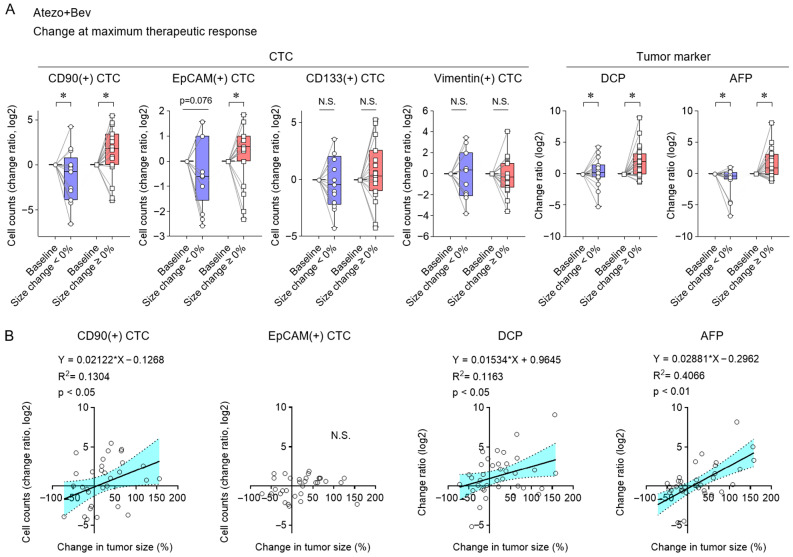
Associations between CTCs and tumor marker changes, as well as maximal tumor response in patients undergoing Atezo + Bev treatment. (**A**) Alterations in CTC subsets and tumor markers at the time of maximal change in tumor size in HCC patients treated with Atezo + Bev, comparing patients who experienced tumor shrinkage (<0%) to those who experienced tumor enlargement (≥0%) from baseline. (**B**) Correlation of maximal changes in tumor size with log2 changes in CD90(+), EpCAM(+) CTC counts, DCP, and AFP levels. A linear regression line is shown with dotted lines indicating the 95% confidence interval. (**A**) Box plots show medians (lines), interquartile ranges (boxes), and ranges (whiskers). Wilcoxon matched-pairs test. (**B**) Simple linear regression analysis. * *p* < 0.05, CI, confidence interval; HR, hazard ratio; N.S., not significant.

In patients with HCC receiving Atezo + Bev, we evaluated changes in CTC counts and tumor markers at maximal therapeutic response. CD90(+) CTCs, DCP, and AFP showed significant alterations corresponding to tumor size changes (Figure 3A), whereas EpCAM(+) CTCs only increased in progressive cases. Changes in CD90(+) CTCs, DCP, and AFP also correlated positively with changes in tumor size (Figure 3B). These findings suggest that changes in CD90(+) CTC counts, DCP, and AFP at the time of maximal response may reflect alterations in tumor size.

### 3.3. Early Changes in CD90(+) CTC Subset and Tumor Markers Associated with Treatment Response Under Lenvatinib Treatment

**Figure 4:**
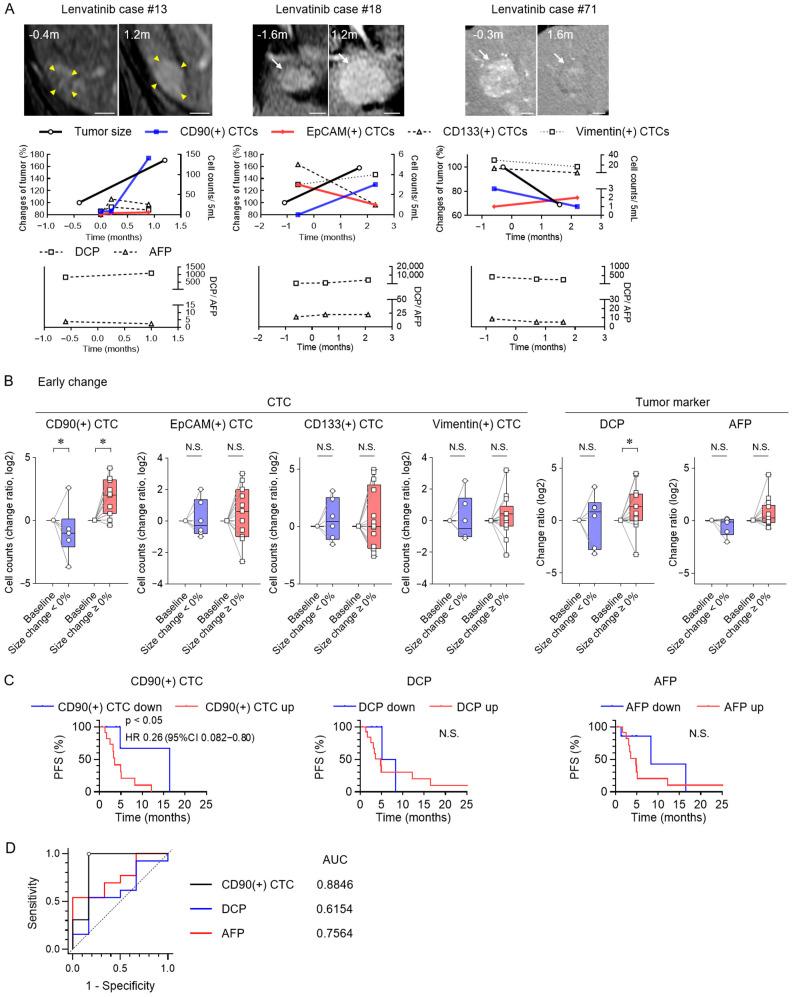
Early changes in CTC subsets and tumor markers predicting outcomes for lenvatinib therapy. (**A**) Representative clinical courses of four HCC patients undergoing lenvatinib therapy. Arterial-phase dynamic CT and MRI images indicate tumor regions (yellow arrowheads or white arrows), along with changes in CTC subsets (CD90, EpCAM, CD133, and vimentin) and tumor markers (DCP and AFP). Scale bar: 10 mm (white bar). (**B**) Early changes in CTC subsets and tumor markers in patients with HCC receiving lenvatinib treatment, comparing groups that experienced tumor shrinkage (<0%) to those that experienced tumor enlargement (≥0%) relative to baseline. (**C**) Kaplan–Meier analysis of PFS according to decreases or increases in CD90(+) CTC counts, DCP, and AFP levels during the early response evaluation. (**D**) Receiver-operating characteristic (ROC) curves of ΔCD90(+) CTCs, ΔDCP, and ΔAFP (log2 ratio) for the prediction of early tumor size changes. (**B**) Box plots show medians (lines), interquartile ranges (boxes), and ranges (whiskers). Wilcoxon matched-pairs test. (**C**) Log-rank test. * *p* < 0.05, AUC, area under the curve; CI, confidence interval; HR, hazard ratio; N.S., not significant.

Longitudinal changes are shown in Figure 4A for three representative patients with unresectable HCC undergoing treatment with lenvatinib. Early alterations in CD90(+) CTC counts were observed in parallel with changes in tumor size after treatment initiation, while AFP and DCP remained largely unchanged during this period. In the full cohort of 19 patients, early changes in tumor size were significantly associated with CD90(+) CTC dynamics (Figure 4B). DCP was only significantly elevated in cases with tumor progression. Early reductions in CD90(+) CTC counts were linked to significantly prolonged PFS, in contrast to AFP and DCP, which showed no such relationship (Figure 4C). ROC analysis of early changes in tumor size showed that the AUC of CD90(+) CTCs was 0.8846 (Figure 4D). The optimal cutoff value for ΔCD90(+) CTCs was −0.390 (log2 ratio), with a sensitivity of 83.3% and a specificity of 100.0% at this threshold. Evaluation of the first treatment response indicated that, in HCC patients treated with lenvatinib, early changes in CD90(+) CTCs correspond to changes in tumor size.

### 3.4. Clinical Implications of CD90(+) CTC Subset and Tumor Marker Changes at Maximal Response for Lenvatinib Therapy

**Figure 5:**
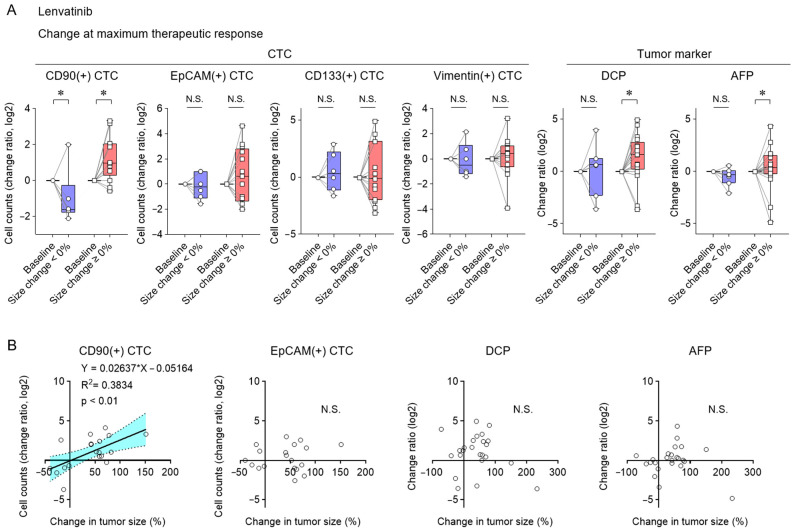
Associations between CTC and tumor marker changes as well as maximal tumor response in patients undergoing lenvatinib treatment. (**A**) Alterations in CTC subsets and tumor markers at the time of maximal changes in tumor size in HCC patients treated with lenvatinib, comparing groups that experienced tumor shrinkage (<0%) to those that experienced tumor enlargement (≥0%) relative to baseline. (**B**) Correlation between maximal changes in tumor size and log2 changes in CD90(+), EpCAM(+) CTC counts, DCP, and AFP levels. A linear regression line is shown with dotted lines indicating the 95% confidence interval. (**A**) Box plots show medians (lines), interquartile ranges (boxes), and ranges (whiskers). Wilcoxon matched-pairs test. (**B**) Simple linear regression analysis. * *p* < 0.05, CI, confidence interval; HR, hazard ratio; N.S., not significant.

Changes in CTC subset counts and tumor markers at the time of maximal treatment response were analyzed in patients with HCC treated with lenvatinib. CD90(+) CTCs underwent significant changes in accordance with tumor size fluctuations (Figure 5A). DCP and AFP levels increased in patients with tumor progression. CD90(+) CTC changes were also positively correlated with changes in tumor size (Figure 5B). These findings suggest that maximal response with lenvatinib therapy enables CD90(+) CTC dynamics to reflect tumor burden.

## 4. Discussion

In this study, we investigated the dynamics of CTC subsets expressing CSC markers at both early treatment stages and maximal therapeutic response in patients with unresectable HCC treated with Atezo + Bev or Lenvatinib. These results were compared with tumor markers AFP and DCP. Changes in CD90(+) CTC counts after Atezo + Bev and lenvatinib treatment were significantly correlated with tumor size changes, PFS, and OS. CD90(+) CTC counts at maximal response also correlated positively with tumor diameter changes. Conversely, early changes in AFP and DCP did not clearly reflect alterations in tumor size. These findings suggest that CD90(+) CTC dynamics are strongly linked to tumor response and prognosis in unresectable HCC when treated with Atezo + Bev or lenvatinib, highlighting their potential as valuable biomarkers.

In this study, alterations in CD90(+) CTC counts following Atezo + Bev and lenvatinib treatment were significantly associated with changes in tumor size, PFS, and OS. Changes in CTC counts have been reported to correlate with therapeutic outcomes in patients with cancer undergoing systemic treatment [10]. We demonstrated previously that analyzing CTCs in patients with HCC is useful for diagnosis, predicting treatment efficacy, and evaluating therapeutic response [14,15,16,17]. In pancreatic cancer [18] and gestational choriocarcinoma [19], variations in CTC counts predicted treatment response more accurately than serum tumor markers. Even in pancreatic cancer patients with normal CA19-9 levels, CTC count changes have been shown to predict outcomes with high sensitivity [18].

CD90 has been identified as a CSC marker in various cancers, including HCC [20,21,22,23]. CD90(+) HCC cells are associated with high metastatic potential and an overexpression of genes related to inflammation, drug resistance, and cell proliferation [23]. CD90 expression is enriched in poorly differentiated HCC and correlates with an unfavorable prognosis. CD90(+) HCC cells exhibit higher malignancy and metastatic ability compared to CD90(−) cells [24,25,26].

Moreover, CD90 expression in HCC cells is induced by TGF-β1 and VEGF, and CD90(+) HCC cells show elevated expression of TGF-β1–related genes [27,28,29]. In our previous analysis, we also observed an increased expression of TGF-β signaling molecules in CTCs derived from HCC patients resistant to Atezo + Bev therapy [16], suggesting that increased CD90(+) CTCs may reflect activation of the TGF-β pathway in resistant cases. In this study, we did not directly evaluate the expressions of VEGF and TGF-β1 in tumor tissues. However, consistent with our previous work [30], prior studies have demonstrated that VEGF levels increase stepwise during the progression from low-grade dysplastic nodules to high-grade dysplastic nodules and subsequently to early HCC [31,32]. Moreover, TGF-β1 has been reported to be more highly expressed in tumor tissues compared with normal liver cells [33]. In addition, HCCs with a high TGF-β signature are associated with pronounced immune exhaustion and indicate a poor prognosis, suggesting that such tumors may exhibit reduced responsiveness to ICI therapy [34,35].

In addition, activation of the WNT/β-catenin pathway has been reported to be a resistance mechanism to ICIs [36] and lenvatinib [37]. In spheroid cultures of HCC cells, elevated β-catenin levels significantly increase CSC markers such as CD90 [38,39]. This suggests that activation of the WNT/β-catenin pathway may also account for the increase in CD90(+) CTCs observed in patients resistant to Atezo + Bev and lenvatinib treatment. Taken together, the emergence of CD90(+) CTCs is not merely an indicator of cancer stemness but also an important biomarker that comprehensively reflects therapeutic resistance and metastatic potential.

Conventional tumor markers, such as AFP and DCP, have been widely reported as useful indicators for evaluating treatment response and prognosis in HCC [40]. A group of HCC patients receiving Atezo + Bev and lenvatinib treatment, AFP levels were assessed 3 weeks after treatment initiation. Longitudinal changes were linked to OS and disease control [41,42,43,44]. However, AFP variations were not informative in patients with low baseline AFP levels [44]. DCP can increase under VEGF inhibitor-induced hypoxia [41,45], meaning that elevated levels may reflect treatment response rather than antitumor effects [46]. In this study, CD90(+) CTCs demonstrated superior dynamic monitoring compared to conventional tumor markers such as AFP and DCP.

This study has several limitations. First, internal validity is compromised by the small sample size and the inclusion of patients from a single hospital without a validation series. To address this limitation, we calculated the detectable effect size in each treatment group based on the number of observed events using Schoenfeld’s approximation. In the Atezo + Bev group (*n* = 37), 26 events were observed for PFS, and hazard ratios (HRs) of ≤0.57 or ≥1.74 were detected with 80% power at α = 0.05. For OS, 23 death events were observed, allowing the detection of HRs ≤0.56 or ≥1.79. The observed HRs were 0.38 for PFS and 0.35 for OS, which are within the detectable range, suggesting that this study has sufficient sensitivity to detect clinically meaningful differences despite the small sample size. In the lenvatinib group (*n* = 25), 17 events were observed for PFS, and HRs ≤0.51 or ≥1.95 were detected with 80% power at α = 0.05. The observed HR for PFS was 0.28 (95% CI, 0.082–0.80), which fell within the detectable range, thereby supporting the robustness of the results in the lenvatinib group as well. Second, a short follow-up time was included for calculating overall survival, which limits the ability to assess long-term outcomes. Therefore, a longer follow-up is required to draw more reliable conclusions regarding the prognostic significance of CD90(+) CTCs. Finally, although multiparametric flow cytometry was sensitive, the low number of detectable CTCs may affect the reliability of our measurements. To establish CTCs as robust biomarkers, large, multicenter prospective studies with appropriate sample size calculation and external validation are required.

## 5. Conclusions

This study found that CSC-related CTC subsets, particularly CD90(+), were more closely associated with tumor response and prognosis in unresectable HCC compared to conventional tumor markers such as AFP and DCP. Our findings highlight the potential value of CTC profiling as a novel approach for disease monitoring and assessing treatment.

## Figures and Tables

**Table 1:** Characteristics of patients with hepatocellular carcinoma treated with atezolizumab plus bevacizumab and lenvatinib.

Characteristics	Atezolizumab + Bevacizumab (*n* = 37)	Lenvatinib (*n* = 25)
Age, median (IQR), years	75 (69–79)	73 (66–78)
Gender, male/female, n	30/7	20/5
ECOG PS, 0/1/2/3/4, n	31/6/0/0/0	23/2/0/0/0
Etiology, HBV/HCV/NBNC, n	3/10/24	3/10/12
PLT, ×10^9^/L, median (IQR)	152 (111–185)	135 (116–163)
PT, INR, median (IQR)	1.05 (0.98–1.15)	1.03 (0.96–1.10)
ALB, g/dL, median (IQR)	3.5 (3.1–3.8)	3.5 (3.5–4.0)
T-bil, g/dL, median (IQR)	0.9 (0.7–1.2)	0.8 (0.6–1.1)
modified ALBI grade, 1/2a/2b/3, n	7/9/19/2	9/4/12/0
ALT, IU/L, median (IQR)	25 (18–38)	29 (14–43)
AFP, ng/mL, median (IQR)	13.7 (4.4–205.4)	7.3 (3.9–90.4)
DCP, mAU/mL, median (IQR)	173 (27–1627)	275 (30–643)
Maximum tumor size, cm, median (IQR)	3.1 (2.5–6.4)	3.4 (2.2–7.7)
Number of tumors, 1/2/3+, n	1/3/33	1/4/20
Vascular invasion, absent/present, n	28/9	19/6
BCLC stage, A/B/C, n	0/22/15	1/15/9
Extrahepatic metastasis, n		
None	28	21
Lymph node	5	3
Bone	1	0
Lung	1	1
Lymph node and Bone	1	0
Lung and Bone	1	0
Prior systemic therapy, n		
None	22	13
Sorafenib	1	0
Lenvatinib	11	―
Atezolizumab plus bevacizumab	―	8
HAIC	1	3
Lenvatinib, HAIC	2	0
Sorafenib and atezolizumab plus bevacizumab	―	1
Observation period, median number of days	324	646

AFP, a-fetoprotein; BCLC stage, Barcelona Clinic Liver Cancer stage; DCP, des-gamma-carboxy prothrombin; HAIC, hepatic arterial infusion chemotherapy; IQR, interquartile range; NBNC, nonB-nonC.

**Table 2:** Antibodies used in this study.

Target	Conjugate	Application	Target Species	Host Species	Clone	Company	Catalog No.
CD45	APC/Cyanine7	Flow cytometry	Human	Mouse	2D1	BioLegend	368,516
pan-Cytokeratin	PE	Flow cytometry	Human	Mouse	C-11	Cayman Chemical	10,478
CD90	PE-Cy7	Flow cytometry	Human	Mouse	5E10	BD Biosciences	561,558
CD133	BV510	Flow cytometry	Human	Mouse	W6B3C1	BD Biosciences	747,644
EpCAM	APC	Flow cytometry	Human	Mouse	EBA-1	BD Biosciences	347,200
Vimentin	Alexa Fluor 488	Flow cytometry	Human	Mouse	RV202	BD Biosciences	562,338

## Data Availability

The data of the current study are available from the corresponding author upon reasonable request. The data are not publicly available due to privacy and ethical reasons.

## Supplementary Materials

The following supporting information can be downloaded at https://www.mdpi.com/article/10.3390/cancers17172829/s1. Figure S1: Study flowchart showing inclusion and exclusion criteria.
