# Supplementary material for: Clinical Significance of CD90(+) Circulating Tumor Cells as Dynamic Biomarkers in Unresectable Hepatocellular Carcinoma Treated with Atezolizumab/Bevacizumab and Lenvatinib
Source: Cancers (Basel). 2025 Aug 29;17(17):2829. doi: 10.3390/cancers17172829 (PMC12427335; doi:10.3390/cancers17172829)
Supplement: Supplementary file 1 [file cancers-17-02829-s001.zip › cancers-3806005-supplementary.pdf]

cancers

MDPI

Supplementary Materials

Clinical Significance of CD90(+) Circulating Tumor Cells as Dynamic Biomarkers in Unresectable Hepatocellular Carcinoma Treated with Atezolizumab/Bevacizumab and Lenvatinib

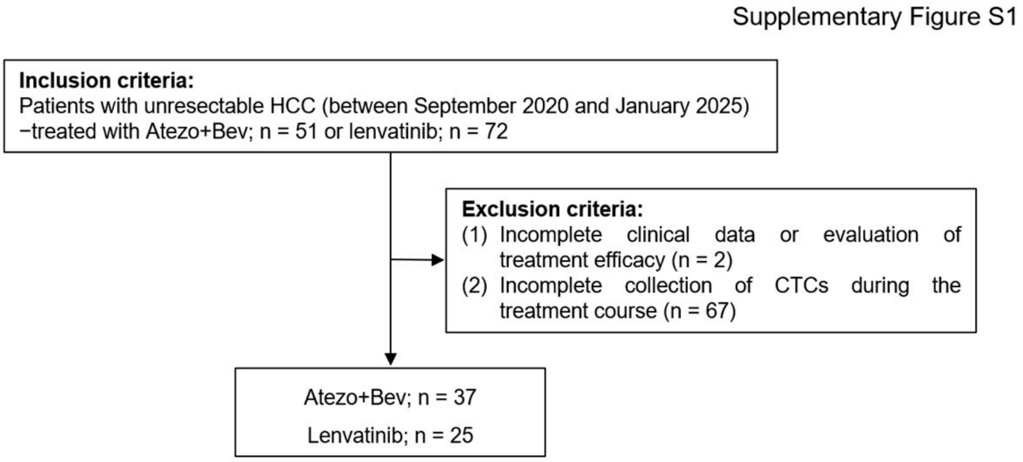

Figure S1. Study flowchart showing inclusion and exclusion.
